# Effects of host sex, age and behaviour on co-infection patterns in a wild ungulate

**DOI:** 10.1017/S0031182025100899

**Published:** 2025-10

**Authors:** Florian Berland, Vincent Bourret, Carole Peroz, Laurence Malandrin, Claire Bonsergent, Xavier Bailly, Sébastien Masseglia, Laurent-Xavier Nouvel, Anne-Claire Lagrée, Clotilde Rouxel, Chloé Dimeglio, Jacques Izopet, Valentin Ollivier, Thierry Boulinier, Isabelle Villena, Dominique Aubert, Vincent Sluydts, Guillaume Le Loc'h, Arnaud Bonnet, Yannick Chaval, Joël Merlet, Bruno Lourtet, Emmanuelle Gilot-Fromont, Hélène Verheyden

**Affiliations:** 1 CEFS, Université de Toulouse, INRAE, Castanet Tolosan, France; 2 LTSER ZA PyGar, Auzeville-Tolosane, France; 3 LBBE, Université de Lyon, CNRS, Villeurbanne, France; 4 Université de Lyon, VetAgro Sup, Marcy-l’Etoile, France; 5 BIOEPAR, INRAE, Oniris, Nantes, France; 6 EPIA, Université Clermont Auvergne, INRAE, VetAgro Sup, Saint-Genès-Champanelle, France; 7 IHAP, Université de Toulouse, INRAE, ENVT, Toulouse, France; 8 BIPAR, Laboratoire de Santé Animale, ANSES, INRAE, ENVA, Maisons-Alfort, France; 9 Laboratoire de Virologie, Centre National de Référence du Virus de l’hépatite E, Toulouse, CHU de Toulouse, INFINITY, Inserm, UT3, France; 10 CEFE, CNRS, Université de Montpellier, EPHE, IRD, Montpellier, France; 11 ESCAPE, Université Reims Champagne-Ardenne, Laboratoire Parasitologie – Mycologie Pôle Biologie – Pathologie, CNR de la Toxoplasmose, CHU Reims, France; 12 University of Antwerp Evolutionary Ecology Group, Wilrijk, Belgium; 13 IHAP, UT3, ENVT, INRAE, Toulouse, France

**Keywords:** behaviour, co-infection, exposure, parasite community, roe deer, susceptibility, wildlife

## Abstract

Recent zoonotic disease emergences emphasize the importance of studying wildlife parasite communities. As wild hosts frequently harbour diverse parasite species, understanding the drivers of multiple infection patterns in free-ranging hosts is critical for elucidating the ecological and epidemiological dynamics of parasite communities. In this study, we analysed co-infection patterns in European roe deer (*Capreolus capreolus*) inhabiting a fragmented rural landscape in southwestern France. Using data from 130 samples of GPS-tracked deer, we examined the influence of proximity to livestock, host activity levels, age, sex and between-parasite interactions on the presence of 11 parasitic taxa. Hierarchical modelling of species communities (HMSC) revealed that proximity to livestock significantly increased the likelihood of infection with orofecally transmitted parasites (*Toxoplasma gondii*, gastrointestinal parasites). Sex and age were other key predictors, with males and juveniles exhibiting a higher frequency of parasite presence, likely influenced by hormonal and immune system differences. Activity levels showed distinct age-related effects, with higher activity levels being positively associated with increased parasite prevalence in yearlings, but not in adults. In contrast, parasite association patterns within individual hosts were weak, suggesting minimal interactions between parasite species. Our findings highlight the interplay between exposure and susceptibility in shaping co-infection patterns and underscore the value of hierarchical modelling approaches in multi-parasite systems.

## Graphical abstract

**Figure:**
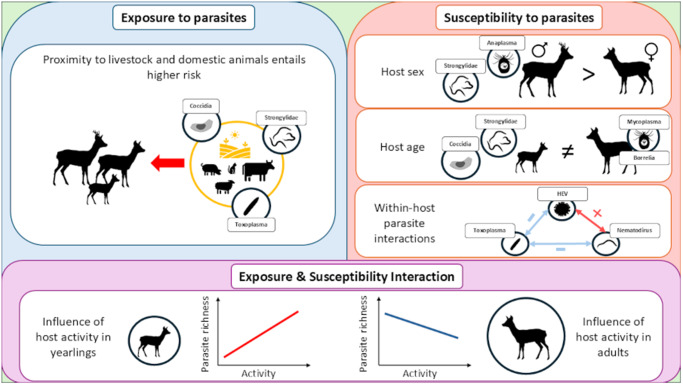

## Introduction

Zoonoses are diseases transmitted between animals and humans and account for over 60% of emerging infections, most (72%) of them originating in wildlife (Jones et al., 2008). This highlights the importance of studying zoonotic pathogens and parasite communities in wild hosts (Smith et al., 2014; Destoumieux-Garzón et al., 2018). We use “parasite” here in the ecological sense, encompassing viruses, bacteria, protozoa and macroparasites. Wildlife hosts are often exposed to multiple parasite species simultaneously (Gortázar et al., 2016; Moutailler et al., 2016; de Cock et al., 2023), leading to potential within-host parasite interactions (Cattadori et al., 2008; Ezenwa, 2016; Keegan et al., 2024). Such interactions may complicate control strategies: for example, deworming treatments in African buffalo increased the spread of bovine tuberculosis (Ezenwa and Jolles, 2015), while in wild mice, nematode removal led to increased protozoan infections due to disrupted parasite competition (Knowles et al., 2013; Pedersen and Antonovics, 2013).

A key target of host-parasite ecological studies is thus to disentangle the effects of between-parasite interactions from other determinants of parasitism influencing the ‘twin pillars of infection’: exposure and susceptibility (Sweeny and Albery, 2022). Exposure represents the likelihood of an individual encountering parasites in its environment, while susceptibility describes the probability of exposure leading to infection (Viney and Graham, 2013; Civitello and Rohr, 2014).

Individual exposure to parasites is influenced by the composition and density of the surrounding host community, which shape parasite and vector abundance, diversity, persistence and transmission dynamics (Arneberg et al., 1998; Parker et al., 2015; Horcajada-Sánchez et al., 2018). High host densities can promote transmission by increasing contact rates, enhancing faecal contamination of the environment, and providing resources for parasite and vector survival (Arneberg et al., 1998). In this context, areas where livestock and wildlife co-occur may represent hotspots of parasite transmission. Having both wild and domestic hosts in shared environments (De Lucia et al., 2018; Horcajada-Sánchez et al., 2018) can increase the likelihood of cross-species transmission and elevate individual exposure to generalist or environmentally persistent parasites (Pato et al., 2013; Sevila et al., 2014; Parker et al., 2015; Verheyden et al., 2020).

Individual behaviour also shapes parasite exposure (Barron et al., 2015, Dougherty et al., 2018). More active or exploratory individuals often show higher infection levels, likely due to increased exposure during movement and foraging (Dunn et al., 2011; Patterson and Schulte-Hostedde, 2011; Bohn et al., 2017; Santicchia et al., 2019). These behaviours may vary with traits like sex, age, or personality, influencing home range size, habitat use and activity patterns (Sih et al., 2004; Bonnot et al., 2018; Malagnino et al., 2021). As a result, behavioural variation can affect (co-)exposure risk (Sih et al., 2018; Brehm et al., 2024) and contribute to individual differences in infection and co-infection patterns, shaping the parasite community within hosts (Fox et al., 2013; Dougherty et al., 2018; Van den Broecke et al., 2021, 2023).

Among other factors, host susceptibility is related to the immune system’s ability to clear parasites, which itself is influenced by individual characteristics such as age, sex, or body condition, through differences in hormone production or resource allocation (Cross et al., 2009). In the context of co-infection patterns, susceptibility to one parasite can also be linked to the presence of other parasites. Parasites differ in their nutritional and energetic needs and in their stimulation of host immunity (Ezenwa and Jolles, 2015; Abbate et al., 2024). Their co-occurrence in a single host can lead to direct or indirect, negative or positive interactions through mechanisms such as immunomodulation or competition between parasites for the host resources (Telfer et al., 2010; Dallas et al., 2019). For example, the presence of gastro-intestinal parasites may enhance the Th2 response and downregulate the Th1 response, thus increasing the susceptibility to some microparasites (Graham, 2008). This situation has been observed in African buffalo, where nematode infection induced Th1 immunity suppression, increasing susceptibility to bovine tuberculosis (Ezenwa et al., 2010). However, the relative impact of within-host parasite interactions and other determinants of immune response such as age, sex and body condition are not clearly established (Van den Broecke et al., 2021, 2023).

To investigate how exposure and susceptibility shape co-infection patterns in wildlife, we analysed individual variation in parasite communities in European roe deer (*Capreolus capreolus*), focusing on the influence of age, sex, behaviour and between-parasite interactions.

Roe deer populations are found throughout most of Europe, exploiting both forested and more open environments, including cultivated lands and livestock breeding areas (Linnell et al., 2020). Such habitat use increases their likelihood for exposure to parasites, including those shared with humans and livestock (Sevila et al., 2014; Beaumelle et al., 2022). Additionally, there is high inter-individual variability in roe deer spatial behaviour, in terms of space use, home range structure and activity (Bonnot et al., 2015, 2018; Malagnino et al., 2021) but strong repeatability for a given individual (Hewison et al., 1998; Gervais et al., 2020; Khazar et al., 2025). Moreover, immune parameters vary among classes of age and sex and with physiological profiles such as stress response in this species (Cheynel et al., 2017; Carbillet et al., 2023). These characteristics make roe deer a relevant biological model for investigating the link between exposure, susceptibility and the composition of parasite communities.

We used data from a roe deer population living in a rural area, where individuals were regularly captured and GPS tracked between 2016–2017 and 2019–2022, and where the presence of 11 parasites have been assessed. We expected several factors to influence the probability of parasite presence in roe deer.

(i) First, following the hypothesis that areas of contact between livestock and wild fauna favour interspecific transmission, we predicted that roe deer using areas where livestock and domestic animals are present should have an increased probability to carry parasites that are shared with domestic hosts (Pato et al., 2013; Sevila et al., 2014). This may concern orofecally transmitted parasites such as Coccidia, *Nematodirus* spp. and Strongylidae (shared with livestock), Hepatitis E virus (excreted by pigs) and *Toxoplasma gondii* oocysts (excreted by cats that may live in proximity to livestock) as well as tick-borne parasites such as *Anaplasma phagocytophilum* (Chastagner et al., 2017).

(ii) Because immunity may differ between sexes and may be less efficient in naïve individuals, we expected that males and young roe deer should be more susceptible to infection and thus showed higher infection probability (Cross et al., 2009; La Peña E et al., 2020).

(iii) Third, because exposure to parasites should increase with increasing levels of exploratory activity, we expected individual activity level to be positively correlated with parasite presence and species richness (Bohn et al., 2017; Santicchia et al., 2019).

(iv) We also considered that the effects of exposure-related factors, such as host activity, may be modulated by individual characteristics like age or sex, which influence susceptibility and shape how exposure translates into infection risk (Sweeny and Albery, 2022). In particular, individuals with high susceptibility, such as juveniles or males (Cross et al., 2009), may experience a stronger effect of activity on their probability of infection.

(iv) Finally, within-host interactions among parasites may modulate infection risk (Dallas et al., 2019). Competition between parasites exploiting the same host resources should promote negative associations in hosts, for instance among tick-borne parasites parasitizing the blood compartment, such as *A. phagocytophilum, Babesia* spp., *Bartonella* spp. and *Mycoplasma* spp. (Telfer et al., 2010). In contrast, positive associations were expected between gastrointestinal macroparasites and microparasites through immunomodulation (Graham, 2008).

## Materials and methods

### Study site and animal monitoring

The study was conducted within the ‘Zone Atelier Pyrénées-Garonne,’ situated in the south-west of France, around the village of Saint-André (43°16 N, 0°51 E). This hilly terrain reaches a maximum altitude of approximately 380 m, and features a mean annual temperature of 12.3°C and an average annual rainfall of 800 mm. The 19 000-ha site encompasses a fragmented rural habitat comprising 2 forested areas and more open environments, including woodlands, hedges, human dwellings, cultivated crops and pasture areas. Landscape permanent structures of the study area have been mapped, and agricultural use is monitored each year at the parcel level as part of long-term ecological studies on this site.

Between January and March from 2016 to 2022, a total of 402 roe deer captures were performed at 6 distinct sites within the study area, representing 319 individuals for which 65 were recaptured at least once. Following capture, the sex was determined, and the age of each roe deer was estimated by assessing tooth condition, classifying individuals into 3 categories: juveniles (6–10 months), yearlings (18–22 months) and adults (older than 2 years). Samples of faeces and whole blood were taken from each captured individual for parasitological and physiological analyses, respectively. Serum was extracted after 10 min of centrifugation at 3000 g. Samples were refrigerated during transport to the INRAE-CEFS laboratory and stored at −20°C (blood and serum) or 4°C (faeces) until analysis.

Prior to release, roe deer were marked with ear-tags and sub-cutaneous microchips. Of the 402 captures, 307 included the fitting of a Global Positioning System (GPS) collar programmed to record at least one position every 6 hours for 48 weeks. The GPS collar was equipped with activity sensors recording acceleration on X (forward/backward) and Y axis (sideways) every 5 minutes (288 records per day) (Benoit et al., 2020).

### Roe deer spatial behaviour

#### Home range

Home range can be defined as the area used by an individual in its normal activities of food gathering, mating and caring for the young (Burt, 1943). Roe deer home ranges were estimated using the Kernel method (Worton, 1989) with the ‘adehabitatHR’ package in R version 4.2.3. We opted for the 95% full home range value to capture most of the area used by the individuals after capture. Because spatial behaviour may be temporarily disturbed after capture (Morellet et al., 2009), the GPS records started 15 days after the date of capture (between January and March) and ended in November (8–10 months later). As roe deer are sedentary and exhibit high spatial fidelity between years (Hewison et al., 1998), the home range observed during the post-capture GPS survey was considered to reflect the usual spatial behaviour and reliably estimate the individual’s exposure to parasite during the period preceding the capture.

However, many juvenile individuals leave their natal area and disperse to another place during the spring following capture (Hewison et al., 1998; Debeffe et al., 2012). In that case, their estimated home range after capture does not represent the spatial behaviour they exhibited before the capture in their natal environment. Thus, we removed from the dataset juvenile individuals that dispersed during the GPS survey (*n* = 63, i.e. 38% of captured juveniles between 2016 and 2022) to avoid misrepresenting true habitat use and exposure during the preceding year.

#### Local species community and livestock presence

The local wild ungulate community is dominated by roe deer and wild boar. Red deer (*Cervus elaphus*) are rare in the area and only occasionally observed. Therefore, their contribution to parasite transmission dynamics in roe deer was considered negligible. Wild boar (*Sus scrofa*) is widely present, but we lack precise data on their spatial distribution within the study area. Nevertheless, due to the high mobility of the species (Morelle et al., 2014) and the number of individuals hunted over all the study area, we assumed that all roe deer were comparably exposed to wild boar.

Land use (woodland, hedgerows, human dwellings, crops and meadows) was mapped every year using a Geographic Information System (GIS; ArcView® 10.0, ESRI Software, Redlands, CA, USA). Pets such as cats and dogs were commonly observed near human dwellings. Half of the meadows were grazed by livestock, mostly cattle (72%), sheep (8%), horses (9%) and rarely goats (<1%) (Chastagner et al., 2017). An indoor pig farm also regularly spreads manure on nearby fields for crop fertilization.

The presence of livestock was monitored weekly in each pasture of the study area throughout the grazing season in 2013 (Verheyden et al., 2020), and in part of the study area in 2021. The areas where pig manure was spread were georeferenced after having questioned the farmer about his agricultural practices. Because we did not have information on local livestock densities in the study area throughout the entire study period and for each species, we chose to index roe deer exposure to livestock using only their presence/absence in the roe deer home range, using the 2013 dataset which is the most comprehensive. We verified that pastoral practices remained stable throughout the study period: 83% of the pastures grazed in both 2013 and 2021 presented the same pastoral practices, suggesting consistency in terms of livestock presence across years. We focused on sheep, cattle and pig manure because previous studies have identified parasite sharing and potential transmission between these livestock species and roe deer, particularly for gastrointestinal parasites (Walker and Morgan, 2014; Verheyden et al., 2020; Beaumelle et al., 2022). Horses and goats were not considered in this study due to their limited relevance, horses sharing few parasites with roe deer, and goats being too sparsely distributed in the study area (4 restricted areas) and typically kept as pets in private gardens. A livestock species was considered present in a roe deer’s home range when the 95% kernel roe deer home range overlapped with a pasture or the area of pig manure spreading. Livestock presence in the roe deer home ranges was then described as a categorical variable with 5 levels: no livestock, cattle, cattle and sheep, cattle and pig manure, and cattle, sheep and pig manure.

#### Mean daily activity

To estimate the level of roe deer activity, we used the mean daily Overall Dynamic Body Acceleration (ODBA, without unit) (Wilson et al., 2006). Dynamic body acceleration is deemed to be a good proxy of energy expenditure (Qasem et al., 2012), notably reflecting movements such as foraging and travelling (Benoit et al., 2020). For every individual, we summed the 288 activity measurements recorded every 5 minutes each day to calculate the total daily activity. We then averaged the daily activity over the entire monitoring period for each individual to obtain the mean daily ODBA value on the overall GPS record period (Khazar et al., 2025). High year-to-year repeatability of individual roe deer activity levels has been highlighted in our population (Khazar et al., 2025), suggesting that individual activity is consistent across the years. As such, we consider mean activity over the monitoring period (starting 15 days after capture and ending the following November) to be a reliable proxy of past activity levels (before capture events).

### Parasite detection

Total genomic DNA was extracted from each whole blood sample using the NucleoSpin® Blood extraction kit (Macherey-Nagel, Düren, Germany). Negative extraction controls were performed by replacing the blood with 200 µL of sterile 1X PBS. Five parasites (*A. phagocytophilum, Babesia capreoli, Babesia venatorum, Bartonella* spp., *Mycoplasma* spp.) were directly detected by PCR of the genomic DNA. The presence and count of eggs were determined from the faeces for gastro-intestinal parasites (Coccidia, *Nematodirus* spp. and Strongylidae) with flotation in hypersaline solution method. For the 3 remaining parasites (*Borrelia* spp., *T. gondii*, hepatitis E virus), assessment of roe deer exposure was performed by antibodies detection in the sera. A recapitulation is available in Table S1 and detailed protocols for each parasite are available in the supplementary material. Due to practical limitations, not all samples could be screened for all the parasites. The detection of *Bartonella* spp. was notably missing for all the samples from 2018.

### Dataset

As the HMSC framework (see below) cannot handle missing data, we worked from a complete dataset obtained from 130 roe deer captures (120 individuals, 10 of which were recaptured once) out of the 307 GPS-collared captures between 2016–2017 and 2019–2022. These captures all met the following criteria: (*i*) the individual was tested for all 11 parasites, (*ii*) at least 75% of its home range was covered by our land-use maps and (*iii*) the individual did not disperse after capture. While this reduced the sample size of our GPS-equipped capture sample, it ensured a comprehensive representation of the parasite community and enabled the analysis of broad patterns of infection across multiple taxa.

### Statistical analysis

To investigate the influence of proximity to livestock, host activity levels, age, sex and parasite interactions on co-infection patterns, we used Hierarchical Modelling of Species Community (HMSC), a recently developed method of hierarchical modelling (Ovaskainen et al., 2017). This method allows one to disentangle the different categories of factors impacting the structure of the studied parasite community (Van den Broecke et al., 2021, 2023).

The HMSC is a joint species distribution model with hierarchical layers, allowing analysis of parasite responses to environmental factors, with the possibility to account for parasite phenotypic traits or phylogenetic distances (Ovaskainen et al., 2017). In our case, explanatory variables related to host exposure (presence of livestock species within the home range, individual activity level) and susceptibility (age and sex) were fixed effects expressed by the β parameter. Moreover, the contribution of the parasite mode of transmission (oro-faecal or vector-borne) to β effects was measured by the γ parameter. We also accounted for potential interactions between parasites by including latent variables that capture residual patterns of parasite co-occurrence not explained by the fixed and random effects. Specifically, we investigated whether parasites tended to co-occur within hosts and years via the Ω variance–covariance matrices. We did not include the phylogenetic distance between the parasites due to the extreme diversity of taxa (bacteria, virus, protozoa) and the different level of identification for each parasite (family, genus, species). The response variable was the binomial presence-absence of each of the 11 parasites (or antibodies for HEV, *Borrelia* spp. and *T. gondii*) in the roe deer. The capture event of individual roe deer was considered as the sampling unit.

To test the effects of the exposure and susceptibility variables, we compared the most complete model to simpler models. The fixed effects included in the most complete model comprised variables related to behaviour (livestock species in the home range, activity level, expected to determine exposure) and host characteristics, i.e. age class (juvenile, yearling or adult) and sex (male or female). As the correlation between activity and the probability of infection was expected to be shaped by host characteristics such as age and sex, we introduced the interactions between activity and both age and sex. To characterize the individual-level structure of parasite communities (within-host parasite co-occurrence) and to account for potential intra-individual similarities in infection profiles across repeated captures (for 10 recaptured individuals), we included individual identity as a random effect. Additionally, we included the year of capture as a random effect, considering that individuals captured in the same year might exhibit more similar infectious profiles, as well as to capture annual variation in the parasite community potentially driven by unmeasured environmental factors. This complete model was compared to 4 simpler models by removing either the interactions, exposure-related variables, susceptibility-related variables or both.

HMSC models were fitted using the R-package ‘Hmsc’ [version 3.0-14] (Tikhonov et al., 2020) with default prior distributions. Posterior distributions were sampled using 4 Markov Chain Monte Carlo (MCMC) chains, each comprising 1 600 000 iterations, with the first 200 000 iterations discarded as burn-in and a thinning of 100 iterations. Each chain yielded 4000 posterior samples, resulting in a total of 16 000 posterior samples. Convergence of the MCMC was assessed using the R-package ‘ggmcmc’ [version 1.5.1.1] (Fernández-i-Marín, 2016), which estimated potential scale reduction factors (Ovaskainen and Abrego, 2020) for model parameters and manual parameter inspection using graphical tools included in the package.

Explanatory and predictive powers were both assessed using the AUC (Pearce and Ferrier, 2000) and Tjur’s R^2^ (Tjur, 2009) values. These measures are based solely on the fixed (β parameters) and random effects of the model and do not account for the latent parameters Ω (parasite co-occurrence structure). Explanatory power was estimated as the proportion of variance in the complete dataset explained by the model parameter average estimates. We then split the dataset randomly into three thirds and calculated the proportion of variance explained by the same global parameter estimates in each of the 3 randomly generated datasets. The average of these 3 proportions was the model predictive power.

Model selection was primarily based on the value of the Tjur’s R^2^ and AUC reflecting explanatory and predictive performance of the model, and secondary on the value of the complementary criterion WAIC (Watanabe, 2010) reflecting the goodness-of-fit in terms of the log posterior predictive score (Ovaskainen and Abrego, 2020). The quality of the model was defined by a high value of Tjur’s R^2^ and AUC and low value of WAIC (Ovaskainen and Abrego, 2020).

We also assessed the relative contributions of fixed and random effects to the variance in infection probability using variance partitioning, which informed the contribution of selected variables (exposure, susceptibility or exposure-susceptibility interaction) on the probability of infection for each parasite (Van den Broecke et al., 2021).

We considered the statistical significance of effects based on the posterior support (PS), i.e. the posterior probability that a parameter is strictly positive or strictly negative (Ovaskainen and Abrego, 2020). For fixed and random effect (β parameter) and parasite trait specific response (γ parameter), we considered an effect to be ‘significant’ when the PS value was ≥0.95, and as showing a ‘tendency’ when it was ≥0.90 but <0.95 (Van den Broecke et al., 2021). For the latent parasite co-occurrence structure (Ω parameter), we followed the approach of Dallas et al. (2019), given the known low statistical power for detecting such interactions in sparse data. We therefore reported all effects with a PS value ≥0.75, to highlight potentially meaningful patterns in co-occurrence or inter-annual structure.

## Results

### Infection and co-infection frequencies

All 130 collected samples showed infection with, or antibodies against, at least one of the eleven parasites analysed. Additionally, 129 of them (99.2%) had been exposed to at least 2 parasites. The most frequent number of parasite taxa detected in a host was 4 (found in 35 individuals, 26.2%), and the maximum number of parasite taxa, found in 2 individuals, was 8 (Fig. S1).

**Figure 1.**
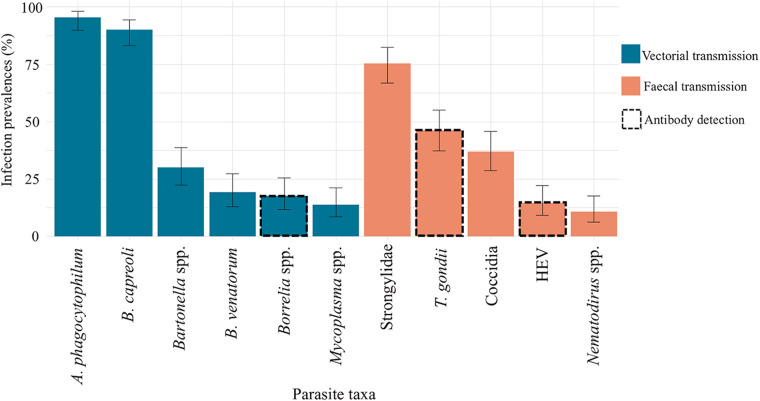
Infection prevalence for 11 parasites in 130 samples from wild roe deer captured between 2016 to 2017 and 2019 to 2022 in South-West France. Error bars represent the 95% confidence interval of the estimated prevalence.

Infection prevalence varied significantly between parasites, from 10.8% for *Nematodirus* spp. to 95.4% for *A. phagocytophilum* (Figure 1).

### Model selection, HMSC convergence and fit

**Figure 2.**
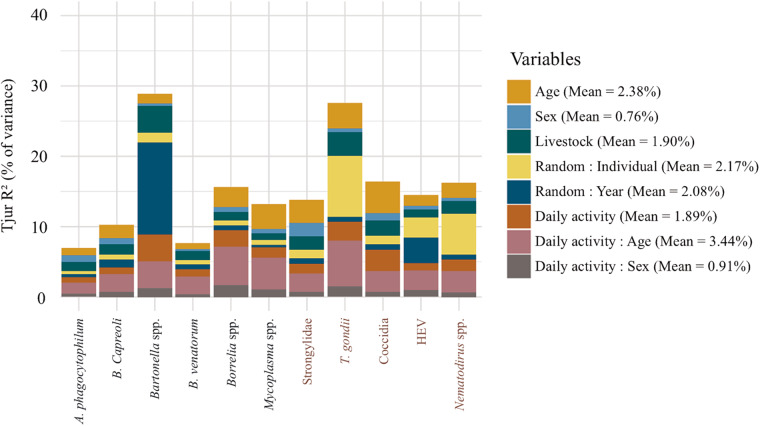
Variance partitioning from the HMSC model showing the proportion of variance (Tjur R^2^) in parasite occurrence explained by fixed and random effects. For each parasite species, the height of each bar represents the relative contribution of each variable to the variance explained. The legend indicates the mean variance explained by each fixed and random effect, averaged across all parasite species. Dark blue coloured parasite names correspond to vector-borne parasites whereas red coloured parasite names correspond to oro-faecal transmitted parasites.

Following model comparison, we kept the most complete model including exposure and susceptibility variables together with interactions between age and activity, and sex and activity. This model had the highest value for the 2 main selection criteria (Tjur’s R^2^ and AUC) for explanatory and predictive performance with the second-lowest WAIC value (Table 1). The WAIC was the lowest for the model with only susceptibility factors and its predictive performance was close to the one of the complete model. Nevertheless, a non-negligible proportion of variance was explained by exposure (3.79%) and exposure-susceptibility interaction (5.33%) variables (Fig. 2), indicating that exposure processes meaningfully contribute to infection patterns, even though their inclusion did not substantially enhance predictive performance. This model was therefore the most appropriate to answer our research question, i.e. to assess the joint effects of exposure and susceptibility factors, and their potential covariation, on the probability of infection and the structure of the parasite community within the host.

**Table 1.** Model characteristics and performance to account for multiple infection patterns in 130 wild roe deer

Model	Random Effects	Fixed Effects	Explanatory mean Tjur R^2^	Explanatory mean AUC	Predictive mean Tjur R^2^	WAIC
Constant	No	No	−0.0001	0.480	−0.006	5.230
Exposure	Year + Individual	Livestock + Activity	0.107	0.814	0.019	5.061
Susceptibility	Year + Individual	Age + Sex	0.101	0.821	0.037	4.925
Exposure & Susceptibility no interaction	Year + Individual	Livestock + Activity + Age + Sex	0.132	0.839	0.039	5.015
Exposure & Susceptibility with interaction	Year + Individual	Livestock + Activity*Age + Activity*Sex	0.155	0.854	0.041	4.994

Visual inspection and potential scale reduction factor evaluation for β, γ and Ω parameters, representing respectively host related variables effect, parasite traits and host related variables association, and parasite associations, confirmed the convergence of the HMSC model. The averaged potential scale reduction factors were estimated to 1.0004 (max = 1.004) for β parameters, 1.0004 (max = 1.003) for the γ parameters, and 1.0016 (max = 1.0117) for the Ω parameters.

The selected model fitted the data with a mean AUC of 0.850 (range: 0.731–0.970) for the explanatory power, and 0.560 (range: 0.29–0.77) for the predictive power. The Tjur R^2^ for the explanatory power was on average 0.155 (range: 0.070–0.288), and 0.041 (range: −0.02–0.09) for the predictive power, meaning that fixed and random effects included in the model explained and predicted the data better than random in average. The 2 most prevalent parasites (*A. phagocytophilum* 95.4% and *B. capreoli* 90%), having low variance, had low Tjur R^2^ (6.7% and 10.1%) which reduced the overall explanatory and predictive performance of the model (averaged over the 11 parasites). Nevertheless, we chose to retain all parasite taxa to obtain a broader view of how host exposure and susceptibility shape parasite community structure, and to detect potential parasite–parasite associations.

### Livestock community in the home range

The mean home range size of roe deer was 246.6 ha, varying from 5 to 1987 ha (95% CI: 150–250). Most home ranges contained pasture used by cattle (93.1%) and some contained sheep pasture (29.2%), or fields spread with pig manure (30.0%) issued from a single pig farm. Some roe deer had home range where 2 (cattle and pigs for 19.2%, cattle and sheep for 18.5%) or 3 domestic species (10.8%) were present.

**Figure 3.**
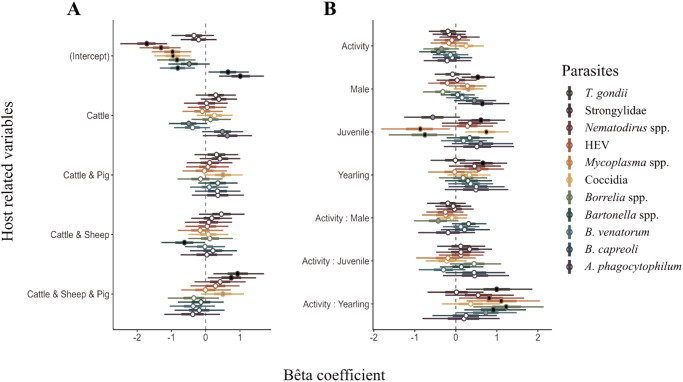
Estimation of beta coefficients for livestock presence (A) and host characteristics (B) on the probability of infection for the 11 tested parasites. The circle on each bar represents the mean of the beta coefficient, and the bar the 95% credibility interval of its posterior distribution. A black circle means the effect is statistically significant (PS ≥ 0.95), a grey circle means the effect is a tendency (PS ≥ 0.90 and < 0.95) and a white means a non-significant effect.

The presence of livestock species in the home range accounted for 1.9% (Fig. 2) of the inter-individual variation in roe deer infection propensity. Roe deer living in home ranges containing the 3 domestic species – cattle, sheep and pig – displayed significantly higher prevalences of *T. gondii* (mean β = 0.93, PS = 0.98) and Strongylidae (mean β = 0.75, PS = 0.96), and tended to be more often infected by Coccidia (mean β = 0.50, PS = 0.91) than roe deer without livestock in their home range. The latter trend also held for roe deer using cattle pasture and pig manure spreading area (mean β = 0.51, PS = 0.93; Fig. 3A). The effect of the presence of the 3 livestock species was associated with the parasite transmission mode, with oro-faecal parasites tending to show a positive response to the 3 species presence (mean γ = 0.56, PS = 0.94; Fig. S3). In addition, roe deer using only cattle pasture tended to be more often infected by *B. capreoli* (mean β = 0.51, PS = 0.93) and *A. phagocytophilum* (mean β = 0.63, PS = 0.93) and less often infected by *Bartonella* spp. (mean β = −0.50, PS = 0.94). Roe deer using cattle and sheep pasture were significantly less often infected by *Bartonella* than roe deer without livestock in their home range (mean β = −0.61, PS = 0.95).

Regarding predicted parasite taxa richness using β parameters, individuals without livestock in their home range have the lower mean parasite richness (mean parasite richness (PR) = 3.97, CI: 2.96–5.11) compared to those with at least one livestock species, such as cattle (mean PR = 4.26, CI: 3.57–5.12), cattle and sheep (mean PR = 4.09, CI: 3.21–5.09), cattle and pig (mean PR = 4.89, CI: 4.08–5.86), or all 3 species (mean PR = 4.81, CI: 3.82–5.94) (Fig. S2). However, this difference in parasite richness was only marked in individuals whose home range included both cattle and pig manure, as the lower bound of their credibility interval exceeded the mean richness estimate of individuals without livestock.

### Age and sex

Roe deer sex and age accounted for 0.76% and 2.38% (Fig. 2) of the variance of parasite infection propensity, respectively. Males had a significantly higher infection rate of *A. phagocytophilum* (mean β = 0.64, PS = 0.96) and Strongylidae (mean β = 0.54, PS = 0.98) and tended to show a higher frequency of infection with Coccidia (mean β = 0.30, PS = 0.94) and *B. capreoli* (mean β = 0.45, PS = 0.93) than females (Fig. 3B).

Among the 3 defined age categories, juveniles were significantly more likely to be infected with gastrointestinal parasites such as Strongylidae (mean β = 0.61, PS = 0.97) and Coccidia (mean β = 0.74, PS = 0.99) and tended to be more frequently infected with *B. capreoli* (mean β = 0.60, PS = 0.92) than adults. In addition, juveniles were less often seropositive to *Borrelia* spp. (mean β = −0.75, PS = 0.96) and less often infected by *Mycoplasma* spp. (mean β = −0.86, PS = 0.98) and tended to be less often seropositive to *T. gondii* compared to adults (mean β = −0.56, PS = 0.92). Yearlings also had a higher probability of infection with Strongylidae (mean β = 0.66, PS = 0.97) and a tendency for a higher prevalence of HEV compared to adults (mean β = 0.57, PS = 0.94). The effect of the yearling age stage on infection probability was associated with the parasite transmission mode, with oro-faecal parasites showing a tendency for a positive response (mean γ = 0.38, PS = 0.90; Fig. S3).

### Roe deer activity

**Figure 4.**
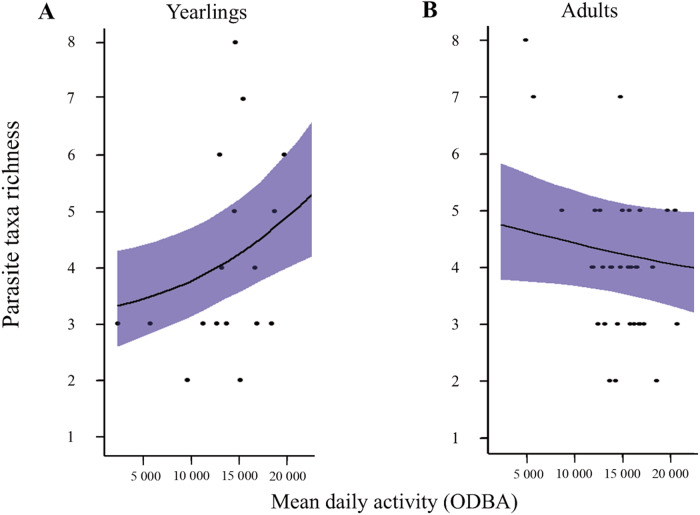
Relationship between host activity and parasite species richness predicted by the HMSC model for yearling and adult roe deer. Sex and presence of livestock species in the home range have been fixed as the more probable values in the dataset (female and cattle). Dots represent observed data; the line represents the predicted mean parasite richness for every activity value and the blue area represents the 95% credibility interval of the estimate.

The level of activity (ODBA) varied between a mean daily value of 1702 to 38 677 in our study data, suggesting strong inter-individual variability. Activity and the interactions between activity and sex and activity and age accounted for 1.89%, 0.91% and 3.44% (Fig. 2) of the variance in parasite infection, respectively. The positive slope of the relationships between activity and infection probability were significantly stronger for yearlings (compared to adults) for *T. gondii* (mean β = 0.99, PS = 0.98), HEV (mean β = 0.81, PS = 0.97), *Mycoplasma* spp. (mean β = 1.10, PS = 0.98), *Borrelia* spp. (mean β = 1.23, PS = 0.99) and *Bartonella* spp. (mean β = 0.92, PS = 0.98) and tended to be stronger for *B. venatorum* (mean β = 0.73, PS = 0.94) (Fig. 4B). The effect of yearling activity on infection probability was related to the parasite transmission mode (Fig. S3). Specifically, vector-borne parasites showed a significant positive response to yearling activity (mean γ = 0.69, PS = 0.96), and parasites transmitted via the oro-faecal contamination tended to respond positively (mean γ = 0.49, PS = 0.90).

This age-dependent effect of activity was also reflected in parasite taxa richness, with a significant positive correlation between activity and parasite richness for yearlings (PS = 0.99) (Fig. 4A). In contrast, in adults, parasite taxa richness almost tended to decrease as host activity increased (PS = 0.89; Fig. 4B).

### Parasite association

Residual parasite co-occurrence after accounting for exposure to domestic animals, activity, age and sex effects, was estimated at the year and individual random effect levels.

**Figure 5.**
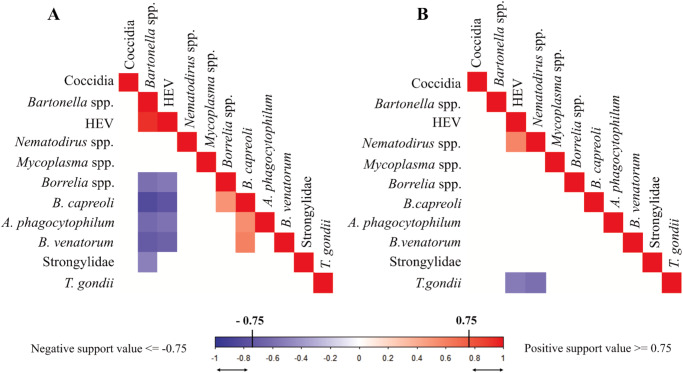
Associations between parasite presences in roe deer, after considering the effects of spatial behaviour, activity and age, sex related effects, at the year level (A) or at the individual level (B). Blue indicates a negative association whereas red indicates a positive association. Colours are shown only for a |posterior support value| > 0.75.

At the year scale, we found a significant positive co-occurrence between HEV and *Bartonella* spp. (mean Ω = 0.18, PS = 0.96), indicating that HEV seroprevalence was strongly correlated with *Bartonella* spp. infection rates (Fig. 5A). We also identified other positive latent co-occurrence patterns with posterior support values above 0.75, including between *B. capreoli* and *Anaplasma phagocytophilum* (mean Ω = 0.02, PS = 0.78), *B. venatorum* (mean Ω = 0.02, PS = 0.81), and *Borrelia* spp. antibodies (mean Ω = 0.01, PS = 0.77).

In contrast, we observed a tendency for negative co-occurrence between both *Bartonella* spp. and HEV with *B. capreoli* (mean Ω = −0.10, PS = 0.93; and mean Ω = −0.04, PS = 0.90, respectively). Other negative associations (PS > 0.75) were found between *A. phagocytophilum* and *Bartonella* spp. (mean Ω = − 0.06, PS = 0.82) or HEV (mean Ω = −0.03, PS = 0.80), and between *B. venatorum* and *Bartonella* spp. (mean Ω =− 0.06, PS = 0.87) or HEV (mean Ω = − 0.03, PS = 0.85), and between *Borrelia* spp. and *Bartonella* spp. (mean Ω = −0.05, PS = 0.81) or HEV (mean Ω = −0.02, PS = 0.79). Similarly, a negative co-occurrence was found between Strongylidae and *Bartonella* spp. (mean Ω = −0.06, PS = 0.82).

At the individual scale, only few associations with posterior support value above 0.75 were revealed. Our model highlighted negative co-occurrence between *T. gondii* antibodies and *Nematodirus* spp. (mean Ω = −0.09, PS = 0.81) or HEV (mean Ω = −0.04, PS = 0.78), whereas HEV antibodies and *Nematodirus* spp. showed positive association (mean Ω = 0.05, PS = 0.80) (Fig. 5B).

## Discussion

As expected, nearly all roe deer were co-infected by several parasites with most often 3–5 parasites among the 11 considered. This result emphasizes the relevance of considering parasite communities and potential parasite–parasite interactions when studying infection patterns (Telfer et al., 2010). The HMSC analysis allowed us to disentangle the effects of the various factors with a satisfactory fit despite the complexity inherent to data recorded in a wildlife population living in a complex environment (Van den Broecke et al., 2021, 2023). As expected, exposure related factors – the presence of livestock and the level of activity – and susceptibility related factors – age, sex and between-parasite interactions – both influenced the co-infection patterns in roe deer. Our approach highlights how host-related factors linked to individual exposure and susceptibility explain part of the variance in infection propensity, while annual environmental fluctuations and weak parasite–parasite interactions may also contribute to the remaining unexplained variance.

### Infection patterns in relation to roe deer exposure to domestic ungulates

Consistent with our first prediction, the presence of several livestock species in roe deer home range increased their likelihood of harbouring parasites that can be shared with domestic animals. Compared to roe deer living in home range without livestock, roe deer moving in areas used by cattle, pig (manure) and sheep exhibited a higher prevalence of Strongylidae and *T. gondii*. A tendency was also detected for Coccidia, which also held in the absence of sheep. These results did not hold with any other livestock species combination. This is likely due to unequal domestic species occurrence across the study area. Pigs (and pig manure) and, to a lesser extent sheep, used a limited space in our study area, whereas cattle are more widely distributed, with most roe deer exploiting their pastures. A quantitative analysis considering the abundance of domestic animals and the intensity of roe deer infection, rather than simple occurrences of domestic animals and parasites, could have revealed a ‘burden intensity effect’ of roe deer exposure to cattle as shown for parasitic helminths in the same area (Verheyden et al., 2020). The lack of information about the quantity of spread manure and about the abundance of livestock over the full parasite sampling period limited our possibility to answer this question. In addition, because the various gastrointestinal parasite species were not easily distinguishable using faecal egg counts, we could not detect more specific host related effects of livestock presence, even though sheep and roe deer are known to share several gastrointestinal parasite species in this area such as *Haemonchus contortus* or *Trichostrongylus axei* (Beaumelle et al., 2022).

Pigs are known to be infected by *T. gondii* (Lehmann et al., 2003; Djokic et al., 2016) and Coccidia (Delsart et al., 2022). However, these parasites are rarely (Coccidia) or never (*T. gondii*) excreted by pigs, reducing the likelihood of interspecific transmission from pig to roe deer. Nevertheless, compared to roe deer that did not use domestic ungulate pastures, individuals with cattle, sheep and pig in their home range are likely to live closer to farms and livestock breeding infrastructures where cats can be abundant. Indirect transmission of cat-derived *T. gondii* oocysts to roe deer, through cat encroachment into roe deer habitat or manure dispersion, could thus explain the significant positive correlation between the occurrence of the 3 domestic ungulate species and the infection by *T. gondii*, as noted in other wild species (Gotteland et al., 2014; Barros et al., 2018). One could also consider a potential effect of wild species present in these areas, such as wild boar or small mammals. However, interspecific transmission between non-felid species is typically linked to cannibalism or predation of small mammals; these behaviours are not relevant in roe deer (Barroso et al., 2020).

Unexpectedly, roe deer using cattle and sheep presence areas exhibit a significant lower prevalence of *Bartonella* spp. Roe deer and cattle can both be infected by *Bartonella bovis* (Skotarczak and Adamska, 2005; Cherry et al., 2009) and we could expect a higher probability of infection for individuals using cattle pastures. However, livestock grazing has been shown to reduce the density of rodents and the abundance of fleas (McCauley et al., 2008; Bueno et al., 2012) which are the main vector of *Bartonella* spp. (Billeter et al., 2008). Thus, the negative correlation found in our model could come from a negative effect of livestock grazing on the abundance of *Bartonella* spp.’s vectors reducing the likelihood of infection for roe deer using these areas.

Nevertheless, we acknowledge that we had no information on the parasitic community hosted by cattle and pigs in our study area, neither in terms of diversity, nor in terms of abundance. It is also possible that various antiparasitic strategies would be used by the breeders leading to local heterogeneity in parasite excretion or in vectorial transmission. Our interpretation of the link between livestock presence and roe deer parasite exposure therefore remains partly speculative. However, we point that the sharing of parasites between roe deer and sheep has been demonstrated in this area (Beaumelle et al., 2022). This highlights potentially fruitful directions for future research, including parasite screening in cattle and pigs to investigate more thoroughly the effects of spatial cohabitation with domestic animals on roe deer exposure to parasites.

### Infection patterns in relation to age and sex

In many mammal species, parasitism has been shown as male-biased (Perkins et al., 2003; Ferrari et al., 2004). These sex-based differences in infection probability are often associated to susceptibility factors such as the immunosuppressive effect of testosterone (Klein, 2000; Foo et al., 2017; La Peña E et al., 2020) or males reduced investment in immunity compared to females (Rolff, 2002) but they also can be associated with exposure factors that can differ depending on the sex such as home range size, activity, or sociability (Brehm et al., 2024). Here, male individuals were significantly more often infected by *A. phagocytophilum* and Strongylidae and tended to be more often infected by Coccidia and *Bartonella* spp. Testosterone is regarded as an immunosuppressive factor that increases the overall probability of infection. The production of androgen hormones may notably reduce the Th2 response (González et al., 2010), which is normally upregulated against gastrointestinal parasite infections (Maizels et al., 2004) and so increase the probability of infection by parasites such as Strongylidae. Concerning the tick-borne parasites, a higher prevalence and intensity of infection have also been found for *Anaplasma marginale* in African buffalo males (Sisson et al., 2023). The authors invoke possible differences in physiology and immunity leading to a higher susceptibility of males compared to females, as shown in a range of mammalian species (Foo et al., 2017). This sex-based difference in tick-borne parasite prevalences can also be due to morphological differences. Being on average 11% heavier than females (Chirichella et al., 2023), male roe deer could host more ticks than females (Kiffner et al., 2011) increasing their exposure to tick-borne parasites. Moreover, males are known to be more active during the reproductive period (Malagnino et al., 2021), which may increase their likelihood of encountering vectors and parasites and thus their probability of infection (Brehm et al., 2024).

Concerning the effect of age, young individuals may not yet have developed acquired immunity, making them more vulnerable to infections requiring a specific immune response (Gasparoni et al., 2003; Body et al., 2011). In our study, younger individuals were more likely infected by Strongylidae (juveniles and yearlings) and Coccidia (juveniles). The Th2 immune response is generally deemed to be effective against helminths (Maizels et al., 2004), and age-related shifts from Th1 to Th2 immunity have been revealed in other mammals (Gardner and Murasko, 2002; Kovacs et al., 2004). Such a shift could also occur in roe deer, causing helminth infection probability to decrease over the growth phase age and the immunity development.

Juveniles were also less infected by 2 vector borne parasites: *Mycoplasma* spp. and *Borrelia* spp. These differences can reflect ticks being more likely to attach to adults due to their larger size (Vor et al., 2010; Kiffner et al., 2011), increasing their exposure to vectorized pathogens compared to juveniles. Moreover, the smaller size of juveniles and their activity, which starts after the peak of nymph abundance in spring, may make them more exposed to larvae, which take their first blood meal (Kahl and Gray, 2023), reducing their probability of infection by parasite without transovarian transmission such as *Borrelia* spp. and *Mycoplasma* spp. (Zhioua et al., 1994; Obiegala et al., 2017).

### Infection patterns in relation to activity

As expected from previous studies (Bohn et al., 2017; Santicchia et al., 2019), the prevalence of several parasites (*T. gondii*, HEV, *Mycoplasma* spp., *Bartonella* spp., *Borrelia* spp., *B. venatorum*) significantly increased with roe deer activity level, but only in yearlings. Parasite taxa richness also increased with activity in yearlings and this correlation tends to reverse at the adult stage.

In our data, a significant proportion of yearlings may have undergone postnatal dispersal during the previous spring, 10 months before (34% of individuals in our population) (Debeffe et al., 2012). This event is stressful and resource-consuming, potentially impacting yearling immunity and susceptibility (Bonte et al., 2012). Moreover, this dispersal exposed yearlings to new environments and parasites, to which they may be more susceptible (Teitelbaum et al., 2018). Consequently, the impact of activity on exposure could be likely more significant for yearlings, leading to a greater number of infections.

However, the opposite correlation between activity and parasite richness found for adults could come from the development of the acquired immunity in the adult stage in consequence of previous exposures (Sweeny and Albery, 2022). The individual repeatability of movement and space use has been highlighted in the studied roe deer population (Gervais et al., 2020; Khazar et al., 2025). More active adults were probably also more active yearlings which were more exposed to parasites during this life stage. As a result, more active adults may have developed a greater and more diversified acquired immunity, making them more resistant to infection (Hawley et al., 2011; Sweeny and Albery, 2022) and reversing the correlation between activity and co-infection. Complementary mechanisms may be involved: the higher activity in adults may reflect a better body condition and so, a higher resistance to infection (Barron et al., 2015; Goossens et al. 2020) or reduced adult roe deer activity may be a consequence of parasitism (Hofmann-Lehmann et al., 2004; Poulin, 2013; McArdle et al., 2018).

This age-dependent effect of activity highlights the necessity of considering both exposure and susceptibility factors and their interactions. Exposure and susceptibility can vary independently or synergistically, depending on numerous ecological factors, which complicate their inference on the probability of infection (Sweeny and Albery, 2022).

### Parasite associations and co-infection patterns

We hypothesized that parasite co-infection patterns might partly result from immunomodulation or resource competition between parasites within the host. However, only a few notable associations were observed (Fig. 5).

*Bartonella* spp. and HEV antibodies showed a positive association at the yearly level, meaning that the presence of one parasite in the roe deer population during a given year was significantly and positively associated with the presence of the other. Moreover, both were negatively associated with tick-borne parasites such as *Anaplasma phagocytophilum, Babesia* species, *Borrelia* spp., and orofecally transmitted Strongylidae. Conversely, tick-borne parasites exhibited positive associations among themselves, suggesting a division between a group of tick-borne parasites and a group comprising *Bartonella* spp. and HEV. The positive association between *Bartonella* spp. and HEV was not due to the few individuals captured in 2019 (*N* = 7), all of which were negative for both parasites, as this trend persisted even when these individuals were excluded from the dataset. This underscores the potential role of year-specific environmental factors, such as temperature, humidity and vegetation, in shaping parasite or vector survival (Storey and Phillips, 1985; Waruiru et al., 2000; Gubler et al., 2001; Turner and Getz, 2010). For example, *Bartonella* spp. and HEV may benefit from increased annual rainfall and humidity, which could boost flea abundance (Gracia et al., 2013), which are the primary vectors of *Bartonella* spp., and increase water flow, facilitating HEV transmission (Tricou et al., 2020). Additionally, recent studies have highlighted competition among ectoparasites (Lutermann et al., 2015). Tick infestations, for instance, have been shown to compete with the occurrence of lice (Hoffmann et al., 2016) and fleas (Krasnov et al., 2010). Consequently, environmental conditions favouring flea proliferation may enhance the transmission of *Bartonella* spp. and HEV outbreaks while reducing tick infestations and the transmission of tick-borne parasites, potentially explaining the patterns observed in our results.

At the individual scale, we did not observe the negative association expected between blood parasites sharing the same resources. We only detected negative association of *T. gondii* with both HEV and *Nematodirus* spp., whereas the 2 later parasites were positively associated. Although some studies suggest that the immunosuppressive effects of hepatitis viruses may enhance helminth infections (Cox, 2001), and that *T. gondii* immunomodulation could reduce microparasite infections (Ahmed et al., 2017), interpreting these results with certainty remains challenging. The slight co-occurrence pattern observed at the individual level indicated that between-parasite interactions in roe deer were likely negligible compared to other factors influencing exposure and susceptibility. Furthermore, these findings may be limited by the dataset, which includes few recaptures and lacks longitudinal monitoring of individuals, thereby restricting the ability to observe changes in parasite communities over time.

## Conclusion

This study highlights the value of integrating exposure and susceptibility factors, along with their interactions, to better understand co-infection patterns in wildlife. By accounting for ecological, behavioural and host-specific characteristics, it provides perspectives on the factors driving inter-individual variability of the parasite community in a complex environment. Such an integrative approach advances our understanding of host-parasite systems and underscores the importance of considering both individual and environmental determinants to unravel co-infection mechanisms. The interplay between exposure and susceptibility factors, such as the influence of livestock presence, host activity, age-specific immune responses and between-parasite interactions, demonstrates the complexity of parasite-host relationships. Future research should expand on this framework by incorporating longitudinal studies to capture temporal dynamics, as well as exploring interactions at a finer scale, such as parasite-specific traits or immune pathways. Quantifying the relative contributions of these factors across different ecosystems could provide deeper insights into generalizable patterns of co-infection. Using recently developed hierarchical methods, this work offers the groundwork for broader ecological investigations into parasite community dynamics, offering a foundation to better understand how environmental, behavioural and physiological factors intersect in shaping parasite communities.

## Supporting information

Berland et al. supplementary material Berland et al. supplementary material

## Supplementary material

The supplementary material for this article can be found at https://doi.org/10.1017/S0031182025100899
